# Effect of Surface Oxidation on Oxidative Propane Dehydrogenation over Chromia: An Ab Initio Multiscale Kinetic Study

**DOI:** 10.1021/acscatal.1c01814

**Published:** 2021-08-24

**Authors:** Matej Huš, Drejc Kopač, David Bajec, Blaž Likozar

**Affiliations:** † Department of Catalysis and Chemical Reaction Engineering, National Institute of Chemistry, Hajdrihova 19, SI-1000 Ljubljana, Slovenia; ‡ Association for Technical Culture of Slovenia (ZOTKS), Zaloška 65, SI-1000 Ljubljana, Slovenia

**Keywords:** dehydrogenation, chromium oxide, surface oxidation, propane, multiscale modeling

## Abstract

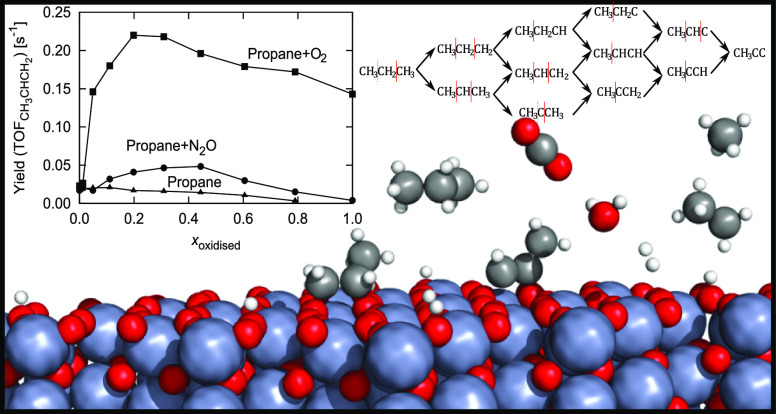

An increasingly utilized way for the production of propene is propane dehydrogenation. The reaction presents an alternative to conventional processes based on petroleum resources. In this work, we investigate theoretically how Cr_2_O_3_ catalyzes this reaction in oxidative and reducing environments. Although previous studies showed that the reduced catalyst is selective for the non-oxidative dehydrogenation of propane, real operating conditions are oxidative. Herein, we use multiscale modeling to investigate the difference between the oxidized and reduced catalyst and their performance. The complete reaction pathway for propane dehydrogenation, including C–C cracking, formation of side products (propyne, ethane, ethylene, acetylene, and methane), and catalyst coking on oxidized and reduced surfaces of α-Cr_2_O_3_(0001), is calculated using density functional theory with the Hubbard correction. Parameters describing adsorption, desorption, and surface reactions are used in a kinetic Monte Carlo simulation, which employed industrially relevant conditions (700–900 K, pressures up to 2 bar, and varying oxidants: N_2_O, O_2_, and none). We observe that over the reduced surface, propene and hydrogen form with high selectivity. When oxidants are used, the surface is oxidized, which changes the reaction mechanism and kinetics. During a much faster reaction, H_2_O forms as a coproduct in a Mars–van Krevelen cycle. Additionally, CO_2_ is also formed, which represents waste and adversely affects the selectivity. It is shown that the oxidized surface is much more active but prone to the formation of CO_2_, while the reduced surface is less active but highly selective toward propene. Moreover, the effect of the oxidant used is investigated, showing that N_2_O is preferred to O_2_ due to higher selectivity and less catalyst coking. We show that there exists an optimum degree of surface oxidation, where the yield of propene is maximized. The coke, which forms during the reaction, can be burnt away as CO_2_ with oxygen.

## Introduction

Short-chained olefins, such as propene (propylene) and butadiene, are important precursor chemicals in the production of plastics, synthetic rubbers, copolymers, epoxides, various organic acids, acrylonitrile, nylon, and so forth. They are predominantly extracted from higher hydrocarbons during steam cracking and fluid catalytic cracking.^1^ Due to the increased demand and environmental concerns, alternative production routes are being developed, among which is dehydrogenation.^2−6^ By utilizing propane as a feedstock, which represents a more prudent use than burning it as fuel, hydrogen is produced when the process is carried out non-oxidatively.^7,8^ However, the high temperatures required due to the endothermicity of the reaction render it less economical. Alternatively, dehydrogenation can be performed with oxidants, such as air, oxygen, N_2_O, or CO_2_, releasing hydrogen as water in a strongly exothermic reaction.

Propane is a rather inert compound (a heat of formation of −104 kJ mol^–1^) with stable C–H bonds (bond-dissociation energy of 410–420 kJ mol^–1^), requiring high temperatures and pressures for activation.^9^ For non-oxidative dehydrogenation to propene and hydrogen, the reaction enthalpy is +124 kJ mol^–1^. For the oxidative dehydrogenation, the reaction enthalpy is −118, −200, or +166 kJ mol^–1^ when using O_2_, N_2_O, or CO_2_ as oxidants, respectively.^10,11^ In addition to water, N_2_ and CO also form.

To steer the reaction toward propene and avoid extensive cracking (non-oxidative) or oxidation (oxidative), appropriate catalysts must be used. In addition to the experimentally^12−16^ and theoretically well-researched alumina-supported Pt/Sn catalysts, which underpin the CATOFIN process and operate at 500–700 °C and 2–4 bar, chromia-based catalysts are also used extensively. Rather than consisting of (semi-)noble metals, they are made of inexpensive source materials. The catalytic performance under optimum conditions (500–600 °C, 1–2 bar) is comparable. Both processes suffer from persistent coking of the catalyst, which must be regenerated or changed often, negatively impacting the catalyst longevity and the process economics.

The use of chromia dates back to the 1930s,^17^ while the first commercial technology was the Pacol process from 1968, using alumina-supported platinum catalysts.^1^ Already in the 1970s, chromium oxide was used for ethylene polymerization.^18^ Soon, the effectiveness of chromia-based catalysts for dehydrogenation had become apparent. Working on chromia-based catalysts, Suzuki and Kaneko already in 1977 proposed a macrokinetic model.^19^ Chang *et al.* showed that doping with Pt improves the performance of α-Cr_2_O_3_(0001) and ZnO(101̅0).^20^ Zhang *et al.* investigated the SBA-15-supported chromia.^21^ Mentasty *et al.* showed that the amount of acidic sites of the alumina support strongly affects the catalytic activity.^22^ Most importantly, Shee and Sayari showed that Cr(III) and Cr(VI) convert back and forth in mesoporous Cr_2_O_3_/Al_2_O_3_ during the reaction.^23^ Gascón *et al.* performed transient kinetic modeling of propane dehydrogenation, which accounted for the coke formation as well.^24^ Chin *et al.* modeled the reaction in an industrial moving bed reactor,^25^ but neither accounted for elementary steps rather than lumped reactions. Nijhuis *et al.* investigated the catalyst surface with *operando* spectroscopic analysis and showed that small amounts of coking in fact improved the activity probably due to improved adsorption of propane.^26^ Gaspar *et al.* showed that depending on the chromium contents, supports, and precursor compounds used, the ratio between the Cr^2+^, Cr^3+^, and Cr^6+^ sites varies, which strongly affects the productivity of the catalyst: Cr^3+^ sites are beneficial for dehydrogenation.^27^

Purely first-principles theoretical descriptions of chromia-based catalysts for propane dehydrogenation remain scarce. In our previous studies, we have investigated the non-oxidative dehydrogenation of propane on the reduced α-Cr_2_O_3_(0001) surface using kinetic Monte Carlo (KMC) on DFT-obtained data^28^ and butane dehydrogenation in an idealized plug flow reactor using DFT-fed microkinetic modeling.^29^ In this work, we used DFT calculations to construct a thorough reaction pathway for propane dehydrogenation when oxidants are co-fed on the reduced and oxidized surfaces of α-Cr_2_O_3_(0001). These are used as the two extrema of the realistic catalyst surface. Using extensive KMC modeling, we investigated the kinetic parameters for propane dehydrogenation on each of the surfaces (temperature and pressure dependence), the effect of varying oxidation state (modeled as a varying ratio between the surfaces), and the effect of the oxidant used (none, O_2_, N_2_O, or CO_2_). We identified the rate-determining steps, the simplified reaction rate law descriptions, and the side products formed. Special emphasis is put on catalyst deactivation, which can manifest as extensive coking (formation of C* and other C-containing surface species), reduction of the catalyst surface, or (not-modeled) sintering and phase transitions.

We show that the oxidized and reduced surfaces behave radically different and how the oxidants influence the selectivity and activity. Although both surfaces bind saturated hydrocarbons weakly, double and triple bond-containing hydrocarbons are strongly adsorbed on the oxidized surface. As a consequence, the latter exhibit a much greater activity but poor selectivity as CO_2_ is mostly produced. Similarly, using O_2_ as a strong oxidant increases the activity and suppresses the selectivity in comparison to using N_2_O. Without the oxidant, the oxidized surface is eventually reduced. Using multiscale modeling, we demonstrate a Goldilocks effect. For optimum conversion of propane to propene, the surface should be partially oxidized. Lastly, we show how the catalyst activity decreases due to coking. Our model captures this as an accumulation of CH_x_^*^ species, which eventually transform into C*. This is, to the best of our knowledge, the first multiscale study of an industrially relevant catalyst in realistic conditions for propane dehydrogenation. Moreover, since the transition between the oxidized and reduced sites is included, this is effectively a model where the catalyst changes during the reaction.

## Computational Details

### Electronic Structure

Theoretical calculations of the electronic structure were performed with VASP^30−33^ in the plane-wave approach. The simulation parameters chosen were consistent with our previous work^28,29^ for comparability. A GGA functional by Perdew and Wang was used (PW91)^34^ with the projector-augmented wave method.^35,36^ To mitigate the self-interaction error on Cr when using GGA functionals, the DFT + *U* approach^37^ was used with a Hubbard factor of *D* – *J* = 4 eV,^38,39^ which had been proved to describe Cr_2_O_3_ satisfactorily.^40,41^ As Cr_2_O_3_ is magnetic, spin-polarized calculations were performed with initial magnetic moments of 3.0 on chromium. The plane waves were expanded to the energy cut-off of 500 eV. The Grimme D3 correction was used to describe the dispersion interactions.^42^

Geometry relaxations were performed with a force threshold of 0.03 eV/Å. For the identification of transition states, the dimer method^43−46^ was used on rough initial approximations from the nudged elastic band method.^47^ All located structures were confirmed with vibrational analysis (a displacement of 0.01 Å) to correspond to local minima (no imaginary frequencies) or saddles (precisely one imaginary frequency) and to obtain the zero-point energies.

Due to the size of the unit cell of Cr_2_O_3_, which was optimized to *a*_0_ = 5.09 Å and *c*_0_ = 13.77 Å, a 4 × 4 × 2 Monkhorst–Pack mesh of *k* points sufficed. The (0001) surface was modeled with 12 layers, of which the bottom six were immovable in their bulk positions. All calculations were performed on a  supercell, where the Brillouin zone was sampled at a Γ point only due to its size (). There was 15 Å of vacuum between the slabs. The dipole correction was used because the slabs were asymmetrical.^48,49^

### Thermodynamics and Kinetics

The reaction mechanism consists of surface reactions (Langmuir–Hinshelwood), reactions on the surface involving gaseous reactants (Eley–Rideal), and adsorption/desorption equilibra, which were modeled within the transition state theory approximation. Among the adsorption reactions, we distinguish simple non-activated adsorptions (for instance, hydrocarbons) and activated dissociative adsorptions (hydrogen, CO_2_, N_2_O, O_2_, *etc.*). The former is a purely kinetic event (eq 3), while the latter is an ER reaction (eq 2). The reaction rates for these types of reactions are calculated as
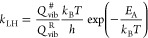
1
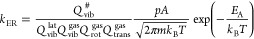
2
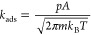
3where *Q*’s stand for partition functions (vibrational from the harmonic approximation, translational, and rotational), *T* for temperature, *E*_A_ for activation barriers, *p* for pressure, *A* for the effective area of the reaction site, and *m* for mass. Furthermore, *k*_B_ denotes the Boltzmann constant and *h* the Planck constant. All the reactions are reversible: *E*_A_fwd__ – *E*_Arev_ = Δ*E* + ΔΔ*E*_lateral_, where ΔΔ*E*_lateral_ denotes the difference between the lateral interaction corrections (Δ*E*_lateral_ = *E*_lattice state_ – *E*_infinite separation_) to the energy of the initial and final states (Δ*E*_lateral final_ – Δ*E*_lateral initial_). The reverse of the non-activated adsorption is treated as an ER-type reaction, with *E*_A_ = Δ*E* = −*E*_ads_.

The adsorption energy is intuitively defined as *E*_ads_ = *E*_slab+adsorbate_ – *E*_adsorbate_ – *E*_slab_, where *E*_slab+adsorbate_ stands for the energy of a relaxed slab with the adsorbate, *E*_adsorbate_ is the energy of the gaseous adsorbate, and *E*_slab_ is the energy of the empty slab. It is decomposed into the electronic interaction, *E*_int_ (negative), and the distortion energies of the adsorbate, *E*_dis_, and surface, *E*_surf,dis_ (both positive). It is evident that *E*_int_ + *E*_dis_ + *E*_surf,dis_ = *E*_ads_.

The reaction energy, Δ*E*, is defined as the difference between the final and initial states (*E*_final_ – *E*_initial_), while for the activation barrier, *E*_A_, the transition state must be known (*E*_TS_ – *E*_initial_). All energies are zero-point energy-corrected.

### Catalyst Model

We model the catalyst as the (0001) surface of Cr_2_O_3_ with 12 layers. To prevent lateral interactions of adsorbates across the adjoining cells, a  supercell is used. Wang and Smith^50^ performed extensive first-principles simulations of this surface and constructed a phase diagram. They showed that five surface phases can exist between two extrema (segregated Cr atoms and condensed oxygen). From the lowest to the highest chemical potential of oxygen, chromium-terminated (A), () 1/9 ML chromyl-terminated (−Cr=O) (B), () 2/9 ML chromyl-terminated (C), (1 × 1) 1/3 ML chromyl-terminated (D), and (1 × 1) 1/3 ML oxygen-terminated (E) surfaces can manifest. Essentially, these different terminations are congruent with different oxidation states of the chromium atoms at the surface. According to the full potential linearized augmented plane wave calculations on the GGA level of theory, Wang and Smith claim that the *E* surface is predominant at 850 K and 1 bar O_2_, which are typical operating conditions for propane dehydrogenation over Cr_2_O_3_ (CATOFIN process).

Several experimental studies have shown that such descriptions of the surface are appropriate. Maurice *et al.* used scanning tunnelling microscopy to show that (1 × 1) differently terminated phases occur as predicted by theory within the error bar.^51^ Rohr *et al.* used low-energy electron diffraction (LEED) and reported a (1 × 1) Cr=O terminated surface, while the reduced phase was discovered below 10^–16^ atm oxygen, which is clearly out of the operating conditions for the reaction at hand.^52^ Petrosyan *et al.* studied Cr_2_O_3_ in solutions using joint DFT and also investigated the oxygen-terminated surface.^53^ In a more recent study, Kaspar *et al.* studied the surface structure of α-Cr_2_O_3_(0001) epitaxial thin films on alumina after activated oxygen exposure using XPS and X-ray photoelectron diffraction (XPD). XPD patterns were found to strongly suggest the Cr–Cr–O_3_ termination.^54^ Similarly, Lübbe and Moritz performed a LEED analysis on α-Cr_2_O_3_(0001) bulk single crystals and found that “for the chromia surface the results indicate that termination with a single Cr seems not to hold”.^55^ Bikondoa *et al.* used XRD to study the surface structure of α-Cr_2_O_3_(0001) and determined that already at an oxygen pressure of 10^–5^ atm, the surface is terminated by chromyl species (−Cr=O).^56^ Although studying a different facet (Cr_2_O_3_(101̅2)), York *et al.* also found the oxygen-termination to predominate.^57^

However, the situation during the reaction, where oxidants (O_2_ and N_2_O) and reducing species (hydrocarbons) are present, is more complicated. First, these conditions are close to a phase transition and a small increase in the temperature or decrease of the oxygen pressure would render the D surface more stable. Second, the accuracy of DFT calculations is limited in terms of chemical accuracy, meaning that surface diagrams can easily be shifted for 100–200 K and several factors for pressure. Third, catalysts are most active near phase transitions. Fourth, while oxygen is an oxidant, propane is a moderate reducing agent. Lastly, in this work, we study the reaction in both, oxidative and non-oxidative regimes, where O_2_ (strong oxidant), N_2_O, and CO_2_ (soft oxidant) of various concentrations or, alternatively, no oxidant are used. This would necessitate taking into account all five surface structures and their interconversion on-the-fly, which adds too many layers of complexity to a simple model.

**Figure 1:**
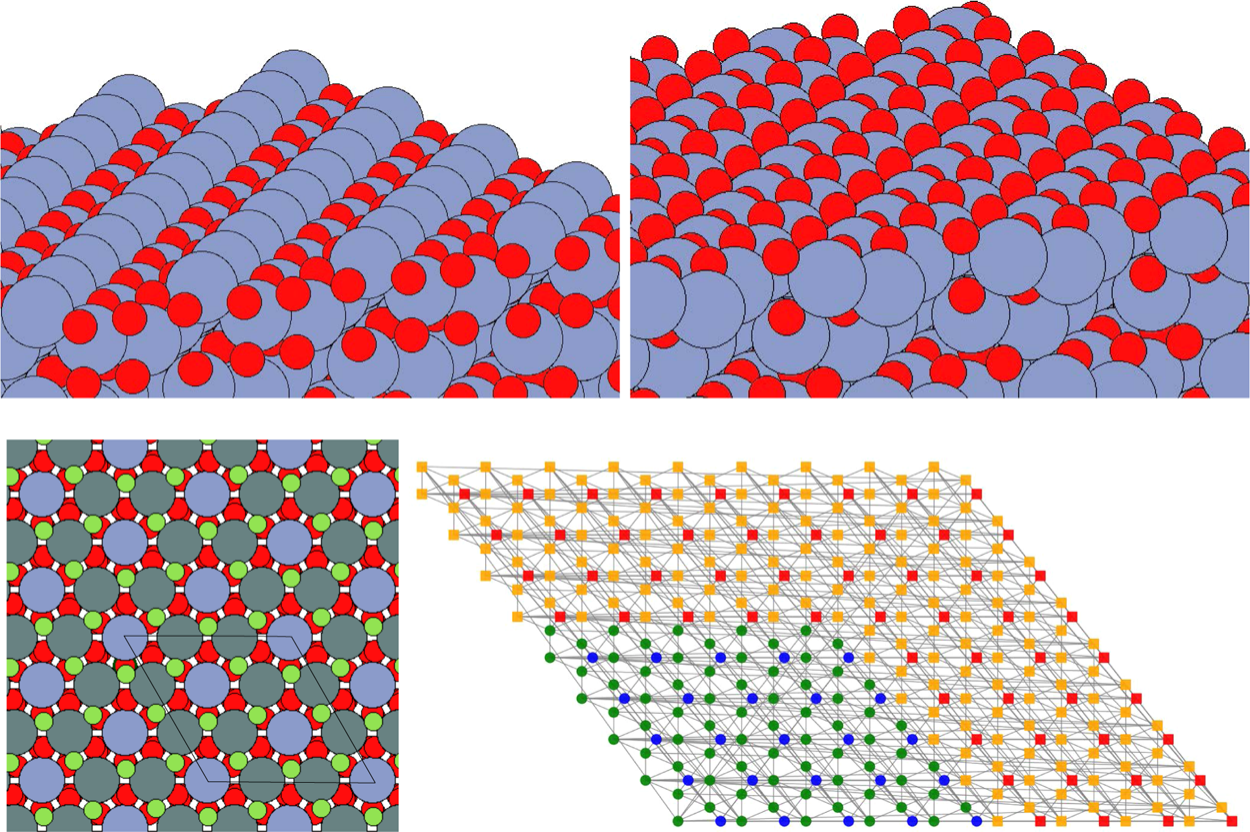
Perspective view of the DFT-optimized (a) reduced (A) and (b) oxidized (E) surfaces Cr_2_O_3_(0001). (c) Top view of the E surface. For the A surface, an additional layer of Cr atoms is situated atop. Color code: red—O, blue—Cr, green—O (top), and teal—Cr (top). (d) An example of the KMC lattice used (sizes vary). Color code: blue—dummy sites, green—O_ox_, red—Cr_red_, and yellow—O_red_.

Noting that the B, C, and D surfaces are essentially the E surface of different surface oxygen (in the form of chromyl) coverages, we construct a simplified model as an alternative. In our model, we use the A surface, which was already used in our previous studies,^28,29^ and the E surface in varying fractions. The model has three types of active sites: chromium atoms on A (Cr_red_), oxygen atoms on A (O_red_), and oxygen atoms on E (O_ox_). The E surface can lose the surface oxygen as O_2_ or, more realistically, H_2_O, being converted to *A*. It, in turn, can be reoxidized to E by N_2_O or O_2_ (see the section Reaction Mechanism for more details). As depicted in Figure 1, the lattice has a quasi-hexagonal symmetry, where each chromium atom is connected to six nearest chromium atoms and three nearest oxygen atoms and *vice-versa*.

### KMC Simulations

The kinetic analysis was performed as KMC simulations. Kinetic and thermodynamics parameters, as obtained from the DFT data, were used in the KMC model to probe the reaction at various temperatures, reactant concentrations (effectively pressures), and catalyst compositions. The simulations were performed using Zacros, which is a graph-theoretical implementation of the KMC approach. In this approach, the Hamiltonian is calculated within the energetic model, accounting for the number of adsorbed clusters on the lattice and their interactions.^58−61^ The raw output from the simulations is the lattice configuration at each time interval and the amount of gaseous species consumed/formed. From these, turnover frequencies (TOFs) and, in turn, apparent activation barriers, reaction order, rate-determining steps, and degree of rate/selectivity control, and so forth are extracted.

**Figure 2:**
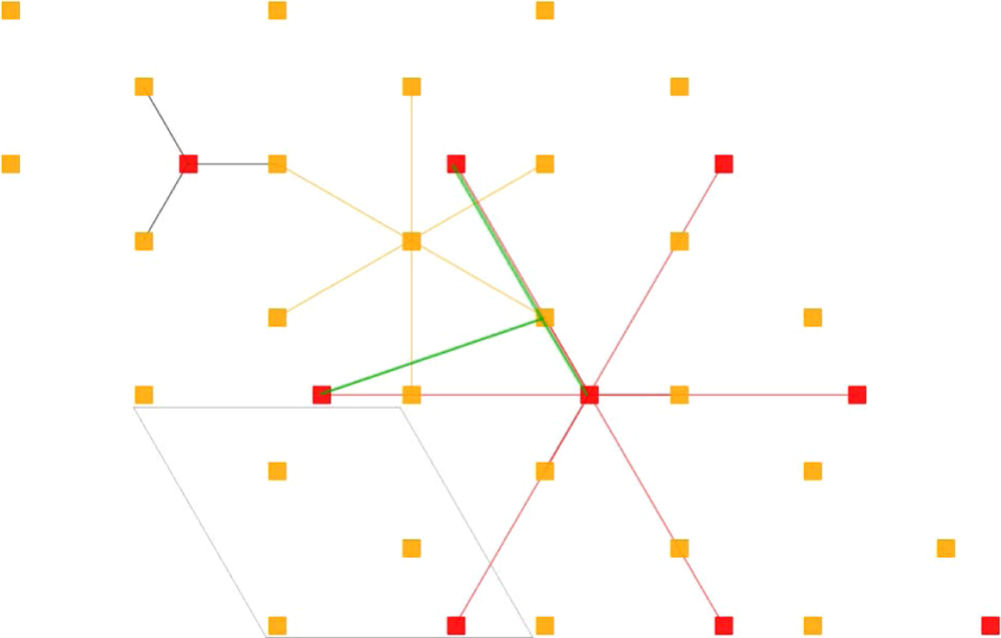
Close-up of the lattice showing the connectivity. A gray parallelogram shows one repeating cell.

The simulations were carried out on a quasi-hexagonal lattice, as shown in Figure 1, which is commensurate with the lattice of the DFT model. There are two types of surface sites: one corresponding to the exposed chromium atoms and the other to oxygen atoms. Each chromium surface atom (site “Cr”) has six neighbors in the hexagonal arrangement. Additionally, each chromium atom connects to three neighboring oxygen atoms (sites “O”). Oxygen atoms are linked to six oxygen atoms in the hexagonal arrangement and the nearest three chromium atoms. In total, each surface site has a connectivity of nine (Figure 2).

To account for the effect of surface oxidation, two different surfaces are investigated: reduced (A) and oxidized (E). The underlying lattice is the same, but the chromium sites are not exposed on the oxidized surface. Thus, three active site types are considered (O_ox_, Cr_red_, and O_red_), while the Cr_ox_ sites in the KMC are considered inert (having no physical counterpart, they are only included to construct the lattice more easily). The reaction constants and, consequently, the reaction mechanism are different on the two investigated surfaces and were individually determined by separate DFT calculations. The KMC simulations were performed on the 12 × 6 lattice, which corresponds to 288 sites. As seen in Figure 1, their relative ratio was varied by changing the size of the E “island” in the bottom left corner.

Preliminary testing showed that quadrupling the lattice does not change the calculated TOFs noticeably (less than 2%). Lateral interactions were calculated for all C_x_^*^–H* pairs up to the first nearest neighbor. The simulations were run with 19 different seeds and then averaged. Adsorption and diffusion reactions were treated as fast equilibrated events (stiffness-scaled).^62^ A typical simulation was terminated after 3 million events, which sufficed to reach a steady performance. Convergence testing of three different simulations (one for each surface type) with 2 million, 3 million, and 4 million events showed that the obtained TOFs differed by less than 1%.

The model is checked to be thermodynamically consistent. All reactions paths on the catalyst (on the oxidized or reduced surface) yield the same reaction energy as is the energy difference between the products and reactants in the gaseous phase. In the kinetic model, two sets of adsorption energies and reaction barriers are available, depending on the oxidation state of the active site.

## Results and Discussion

### Adsorption

Saturated hydrocarbons, of which methane, ethane, and propane were included in the model, interact with the surfaces merely through weak and non-specific van der Waals interactions. This results in high barriers for their activation (see the section Reaction Mechanism and Table 4), low surface coverages during the reaction, and negligible surface perturbation. As summarized in Table 1, the adsorption is weaker on the oxidized surface (0.23 eV *vs* 0.36 eV for propane, 0.21 eV *vs* 0.23 eV for ethane, and 0.11 eV *vs* 0.14 eV for methane), whereas the surface and adsorbate distortion energy due to perturbation is negligible. Molecular hydrogen does not bind to the surface. The attractive interaction is wholly due to electronic effects being weaker on the oxidized surface due to the higher electron density on the surface, repelling the saturated hydrocarbons. A Bader charge analysis shows that on the oxidized surface, oxygen atoms with a Bader charge of −0.68 are exposed, whereas the reduced surface is terminated with chromium atoms with a charge of +1.56, while the three neighboring oxygen atoms have a charge of −1.05. Effectively, the oxidized surface exhibits an acidic character with exposed oxygen atoms, which readily take on hydrogen atoms.

**Table 1:** Adsorption Energies for Stable Compounds in the Reaction Scheme Can Be Decomposed Into the Interaction and Distortion Energy, Such That *E*_surf,dis_ + *E*_dis_ + *E*_int_ = *E*_ads_^a^

	reduced surface (A)				oxidized surface (E)			
species	*E*_surf,dis_	*E*_dis_	*E*_int_	*E*_ads_	*E*_surf,dis_	*E*_dis_	*E*_int_	*E*_ads_
C_3_H_8_	0.00	0.02	–0.38	–0.36	0.01	0.01	–0.25	–0.23
CH_3_CH=CH_2_	0.03	0.02	–0.50	–0.45	1.20	2.68	–6.88	–3.00
CH_3_C≡CH	0.04	0.02	–0.69	–0.63	3.40	3.59	–11.09	–4.10
C_2_H_6_	0.00	0.02	–0.25	–0.23	0.00	0.00	–0.21	–0.21
CH_2_=CH_2_	0.02	0.02	–0.43	–0.39	1.16	2.45	–6.50	–2.89
CH≡CH	0.04	0.02	–0.46	–0.40	2.78	3.26	–10.23	–4.19
CH_4_	0.00	0.01	–0.15	–0.14	0.00	0.00	–0.11	–0.11
H_2_	0.00	0.00	–0.04	**–0.04**	0.00	0.00	0.00	**0.00**

a All values are in eV.

Hydrocarbons with multiple bonds (propene, propyne, ethene, and ethyne) react with the two surfaces differently. While on the reduced surface they bind to an exposed chromium atom by the interaction of their π electron cloud, they bind with the sp^2^ or sp carbon atoms to oxygen atoms of the oxidized surface. The latter interaction is approximately 1 order of magnitude stronger, accompanied by the strong electronic interaction and geometric effect. For instance, propene adsorbs strongly (−3.00 eV) despite the large distortion energies of the surface (+1.20 eV) and adsorbate itself (+2.68 eV). Such strong interactions have a profound effect on the reaction selectivity as the intermediates do not readily desorb but instead undergo further dehydrogenation or cracking, as shown later on.

KMC simulations offer insight into the behavior of the catalyst structure with an atomistic resolution provided sufficient input data are available. Lateral interactions are key to transcending a mean-field description. In Table 2, we list lateral interactions between the co-adsorbed H* and every other intermediate in the reaction network. This was shown to be sufficient in our previous work,^28^ while including all possible lateral interactions is impractical due to the sheer number of combinatorial possibilities. With a few exceptions, these interactions are weaker on the oxidized surface, where they are also generally repulsive.

**Table 2:** Lateral Interactions (in eV) on the Reduced (A) and Oxidized (E) Surfaces

species I	species II	*E*_int_(A)	*E*_int_(E)
H	H	–0.24	–0.05
C_3_H_8_	H	+0.01	0.00
CH_3_CH_2_CH_2_	H	+0.08	+0.20
CH_3_CHCH_3_	H	+0.06	+0.06
CH_3_CH_2_CH	H	–0.01	+0.25
CH_3_CHCH_2_	H	+0.03	+0.00
CH_3_CCH_3_	H	–0.10	+0.05
CH_3_CH_2_C	H	–0.25	+0.04
CH_3_CHCH	H	+0.07	+0.37
CH_3_CCH_2_	H	+0.03	–0.18
CH_3_CHC	H	–0.36	+0.23
CH_3_CCH	H	+0.02	–0.39
CH_3_CC	H	–0.27	+0.37
C_2_H_6_	H	0.00	0.00
CH_3_CH_2_	H	+0.06	+0.07
CH_3_CH	H	–0.24	–0.01
CH_2_CH_2_	H	+0.02	+0.07
CH_3_C	H	–0.81	+0.18
CH_2_CH	H	–0.06	+0.24
CH_2_C	H	–0.26	+0.42
CHCH	H	+0.08	+0.25
CHC	H	–0.27	+0.20
CC	H	–1.04	+0.06
CH_4_	H	0.00	0.00
CH_3_	H	+0.02	+0.11
CH_2_	H	–0.50	+0.07
CH	H	–0.48	+0.18
C	H	–0.89	+0.06

### Surface (Re)-Oxidation

**Figure 3:**
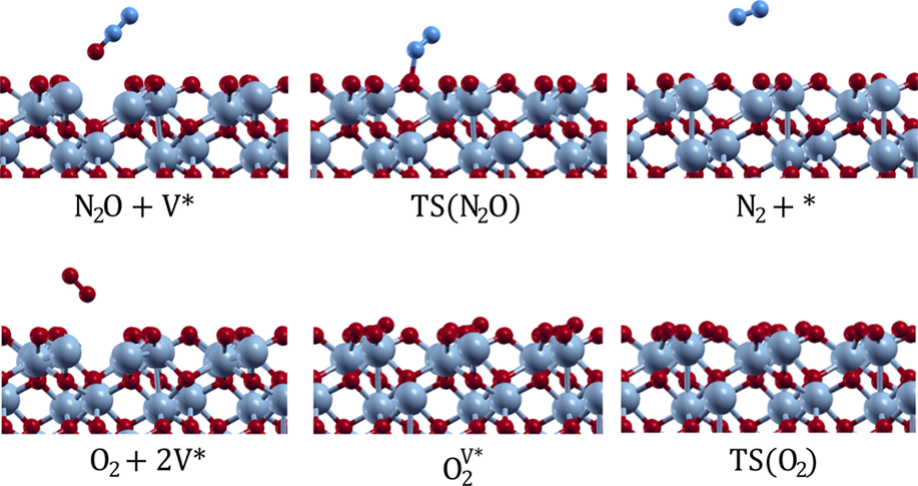
Structures involved in reoxidation of the surface (A to E), labeled as in Table 3.

On the reduced surface, hydrogen atoms bound to oxygen atoms can only recombine into H_2_ and desorb.^28,29^ However, the oxidized surface can lose its surface oxygen as it gets reduced. Recombination of hydrogen atoms on two adjacent oxygen surface atoms yields chemisorbed water, which can desorb. The ensuing oxygen vacancy can migrate across the surface with a kinetic barrier of 0.63 eV (see reaction 3 in Table 3). This migration is limited to the oxidized part of the catalyst (this is relevant only in the mixed composition). If all surface oxygen atoms are lost, the oxidized surface (E) is equivalent to fully reduced (A). The oxygen vacancy can be replenished by N_2_O in an exothermic reaction, yielding N_2_ and the fully oxidized surface. The use of CO_2_, however, is calculated to be less effective due to a high barrier and strong endothermicity. When two adjacent oxygen vacancies form, they are easily filled by dissociative adsorption of O_2_. This reaction is two-step. First, O_2_ strongly adsorbs near the vacancies and then it dissociates. These reactions, effectively enabling a transformation between A and E, are summarized in Table 3 and included in the kinetic model. See Figure 3 for the structures involved.

**Table 3:** Reaction Steps Involved in the Oxidation Interconversion between the Reduced and Oxidized Surfaces^a^

	reaction	*E*_A_ (eV)	Δ*E* (eV)^b^
1	2H* → H_2_O_surf_^*^ + *	1.19	+0.91
2	H_2_O_surf_^*^ → H_2_O(g) + Vac*	1.36	+1.36
3	Vac* + * → * + Vac*	0.63	+0.00
4	Vac* + N_2_O(g) → * + N_2_(g)	0.73	–1.32
5	Vac* + CO_2_(g) → * + CO(g)	2.73	+2.35
6	2Vac* + O_2_(g) → O_2_^Vac*^	0.00	–2.52
7	O_2_^Vac*^ → 2*	0.64	–1.41

a Asterisks (*) denotes empty sites on the oxidized surface (O_ox_), while oxygen vacancies (Vac*) correspond to the motif of the reduced surface.

b Reaction energies are relative to infinitely separated reactants and/or products.

The surfaces (A) and (E) represent two extrema. During the reaction, the catalyst exists in an intermediate state, which is characterized by the fraction of oxygen vacancies (V*) on the surface (E).

### Reaction Mechanism

The reaction mechanism for propane dehydrogenation, although consisting of 74 individual steps, is composed of only four types of reactions (adsorption, ER reactions, diffusion, and Langmuir–Hinshelwood reactions). The steps with the corresponding barriers and reaction energies are listed in Table 4. Propane, propene, propyne, ethane, ethene, ethyne, and methane can adsorb (*vide supra*), while molecular hydrogen interacts weakly and non-specifically with either surface (steps 8–15). However, it readily dissociates and binds as H(−O) (step 16) and diffuses across the surface (step 17). The carbon species either bind too strongly (all unstable intermediates and multiple bond-containing species on the oxidized surface), rendering them immobile, or too weakly, making diffusion comparable with desorption. The difference in the acidity of the surfaces is noticeable, as the hydrogen adsorption energy is −1.69 eV on the oxidized surface and −0.14 eV on the reduced surface (relative to 1/2H_2_(g)).

**Table 4:** ZPE-Corrected Activation Barriers and Reaction Energies for the Elementary Reactions in the Model^a^

			reduced surface (A)		oxidized surface (E)	
	reaction step	type	*E*_A_	Δ*E*^b^	*E*_A_	Δ*E*^b^
8^&^	H_2_(g) + 2# → H_2_^##^	ads.	0	–0.04	0	0.00
9^&^	C_3_H_8_(g) + * → C_3_H_8_^*^	ads.	0	–0.37	0	–0.23
10^&^	CH_3_CH=CH_2_(g) + * → CH_3_CHCH_2_^*^	ads.	0	–0.45	0	–3.00
11^&^	CH_3_C≡CH(g) + * → CH_3_CCH*	ads.	0	–0.61	0	–4.10
12^&^	CH_3_CH_3_(g) + * → CH_3_CH_3_^*^	ads.	0	–0.23	0	–0.21
13^&^	CH_2_=CH_2_(g) + * → CH_2_CH_2_^*^	ads.	0	–0.39	0	–2.89
14^&^	CH≡CH(g) + → CHCH*	ads.	0	–0.40	0	–4.19
15^&^	CH_4_(g) + * → CH_4_^*^	ads.	0	–0.14	0	–0.11
16	H_2_^##^ → 2H^#^	dis.	0.54	–0.24	0.58	–3.38
17^&^	H^#^ + # → # + H^#^	diff.	0.61	0	0.94	0
18	C_3_H_8_^*^ + # → CH_3_CH_2_CH_2_^*^ + H^#^	dehydr.	1.25	+0.85	0.19	–2.64
19	C_3_H_8_^*^ + # → CH_3_CHCH_3_^*^ + H^#^	dehydr.	1.27	+0.73	0.11	–2.70
20	CH_3_CH_2_CH_2_^*^ + # → CH_3_CH_2_CH* + H^#^	deep	1.88	+1.59	0.55	–1.88
21	CH_3_CH_2_CH_2_^*^ + # → CH_3_CHCH_2_^*^ + H^#^	dehydr.	1.37	+0.04	1.76	–2.27
22	CH_3_CHCH_3_^*^ + # → CH_3_CHCH_2_^*^ + H^#^	dehydr.	0.84	+0.16	0.69	–2.21
23	CH_3_CHCH_3_^*^ + # → CH_3_CCH_3_^*^ + H^#^	deep	1.74	+1.44	3.57	–2.08
24	CH_3_CH_2_CH* + # → CH_3_CH_2_C* + H^#^	deep	1.87	+1.62	0.60	+0.45
25	CH_3_CH_2_CH* + # → CH_3_CHCH* + H^#^	deep	1.79	–0.64	0.21	–2.16
26	CH_3_CHCH_2_^*^ + # → CH_3_CHCH* + H^#^	dehydr.	1.42	+0.90	2.14	–1.77
27	CH_3_CHCH_2_^*^ + # → CH_3_CCH_2_^*^ + H^#^	dehydr.	1.22	+0.82	0.23	–1.90
28	CH_3_CCH_3_^*^ + # → CH_3_CCH_2_^*^ + H^#^	deep	0.64	–0.46	0.20	–2.03
29	CH_3_CH_2_C* + # → CH_3_CHC* + H^#^	deep	0.30	–0.59	0.21	–2.34
30	CH_3_CHCH* + # → CH_3_CHC* + H^#^	deep	1.98	+1.68	2.40	+0.27
31	CH_3_CHCH* + # → CH_3_CCH* + H^#^	dehydr.	1.81	+0.37	0.96	–0.99
32	CH_3_CCH_2_^*^ + # → CH_3_CCH* + H^#^	dehydr.	1.31	+0.45	0.83	–0.86
33	CH_3_CHC* + # → CH_3_CC* + H^#^	deep	0.86	–0.62	0.35	–0.90
34	CH_3_CCH* + # → CH_3_CC* + H^#^	deep	0.92	+0.69	0.95	–0.36
35	C_3_H_8_^*^ + * → CH_3_CH_2_^*^ + CH_3_^*^	cracking	3.23	+1.23	3.02	–2.41
36	CH_3_CH_2_CH_2_^*^ + * → CH_3_CH_2_^*^ + CH_2_^*^	cracking	2.90	+1.92	1.96	–1.11
37	CH_3_CH_2_CH_2_^*^ + * → CH_3_^*^ + CH_2_CH_2_^*^	cracking	2.32	+0.60	3.15	–1.79
38	CH_3_CHCH_3_^*^ + * → CH_3_CH* + CH_3_^*^	cracking	2.95	+2.22	1.83	–1.58
39	CH_3_CHCH_2_^*^ + * → CH_3_^*^ + CH_2_CH*	cracking	3.29	+1.44	1.96	–1.28
40	CH_3_CHCH_2_^*^ + * → CH_3_CH* + CH_2_^*^	cracking	N/A	N/A	0.92	–0.71
41	CH_3_CCH_3_^*^ + * → CH_3_C* + CH_3_^*^	cracking	2.55	+2.16	N/A	N/A
42	CH_3_CH_2_CH* + * → CH_3_^*^ + CH_2_CH*	cracking	3.20	–0.11	2.81	–1.67
43	CH_3_CHCH* + * → CH_3_^*^ + CHCH*	cracking	2.79	+1.26	2.30	–0.52
44	CH_3_CCH_2_^*^ + * → CH_3_^*^ + CH_2_C*	cracking	3.03	+2.24	N/A	N/A
45	CH_3_CH_2_C* + * → CH_3_^*^ + CH_2_C*	cracking	2.76	–0.11	1.64	–1.76
46	CH_3_CCH* + * → CH_3_^*^ + CHC*	cracking	3.14	+1.46	N/A	N/A
47	CH_3_CHC* + * → CH_3_^*^ + CHC*	cracking	3.13	+0.16	2.66	–0.29
48	CH_3_CHC* + * → CH_3_CH* + C*	cracking	N/A	N/A	0.70	+0.22
49	C_2_H_6_^*^ + # → CH_3_CH_2_^*^ + H^#^	dehydr.	1.42	+0.76	0.28	–2.66
50	CH_3_CH_2_^*^ + # → CH_2_CH_2_^*^ + H^#^	dehydr.	1.42	+0.21	0.92	–2.01
51	CH_3_CH_2_^*^ + # → CH_3_CH* + H^#^	deep	1.99	+1.72	0.43	–1.87
52	CH_2_CH_2_^*^ + # → CH_2_CH* + H^#^	dehydr.	1.28	+0.88	0.36	–1.77
53	CH_3_CH* + # → CH_3_C* + H^#^	deep	1.59	+1.83	0.64	+0.39
54	CH_3_CH* + # → CH_2_CH* + H^#^	deep	0.60	–0.63	0.13	–1.91
55	CH_2_CH* + # → CH_2_C* + H^#^	deep	1.86	+1.63	1.33	+0.36
56	CH_2_CH* + # → CHCH* + H^#^	dehydr.	1.47	+0.72	0.13	–1.01
57	CH_3_C* + # → CH_2_C* + H^#^	deep	0.17	–0.83	0.04	–1.94
58	CHCH* + # → CHC* + H^#^	deep	0.70	+0.58	1.10	+0.51
59	CH_2_C* + # → CHC* + H^#^	deep	0.55	–0.32	0.26	–0.69
60	CHC* + # → CC* + H^#^	deep	1.99	+3.04	1.07	+0.92
61	C_2_H_6_^*^ + * → CH_3_^*^ + CH_3_^*^	cracking	3.13	+1.11	2.81	–2.23
62	CH_3_CH_2_^*^ + * → CH_3_^*^ + CH_2_^*^	cracking	2.75	+1.89	2.25	–0.91
63	CH_2_CH_2_^*^ + * → CH_2_^*^ + CH_2_^*^	cracking	N/A	N/A	1.05	–0.23
64	CH_3_CH* + * → CH_3_^*^ + CH*	cracking	2.53	+2.27	N/A	N/A
65	CH_3_C* + * → CH_3_^*^ + C*	cracking	2.30	+2.03	1.59	–1.30
66	CH_2_C* + * → CH_2_^*^ + C*	cracking	N/A	N/A	0.26	–0.69
67	CHC* + * → CH* + C*	cracking	N/A	N/A	1.12	+0.66
68	CC* + * → C* + C*	cracking	N/A	N/A	0.45	–2.61
69	CH_4_^*^ + # → CH_3_^*^ + H^#^	deep	1.42	+0.78	0.48	–2.46
70	CH_3_^*^ + # → CH_2_^*^ + H^#^	deep	1.98	+1.54	0.64	–1.34
71	CH_2_^*^ + # → CH* + H^#^	deep	2.31	+2.11	0.69	+0.48
72	CH* + # → C* + H^#^	deep	1.86	+2.01	1.09	–2.71
73	C* + * → CO_2_(g) + 2Vac*	burn			0.96	–1.60
74	C* + O_2_(g) → CO_2_(g) + *	burn	1.12	–3.95	0.96	–2.22

a Asterisks (*) and hash signs (#) denote the Cr_red_ and O_red_ sites on the reduced surface (A). Data for the reduced surface are from ref (28). On the oxidized surface (E), only O_ox_ sites are available, binding *all* intermediates competitively. Fast-equilibrated steps are indicated by the ampersand sign (&).

b Reaction energies are relative to infinitely separated reactants and/or products. N/A denotes cracking reactions that are deemed inaccessible on a given surface due to excessive reaction energy (Δ*E* > 3.0 or Δ*E* > 1.0 eV on the reduced and oxidized surfaces, respectively).

**Figure 4:**
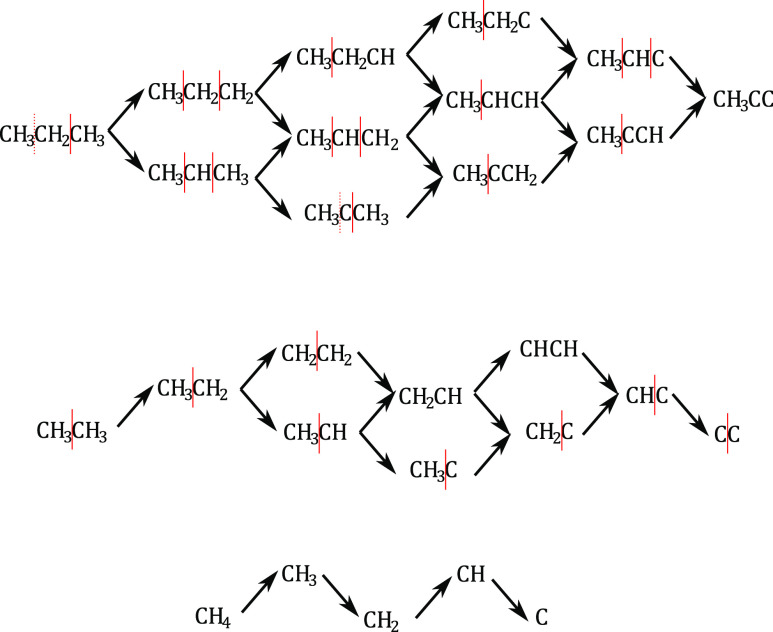
Schematic representation of the reaction mechanism with all carbon intermediates. Black arrows represent (de)hydrogenation reaction steps (all reactions are considered reversible in the model) and red lines represent a possible C–C cleavage. Dotted lines represent cleavages that are accounted for due to symmetry.

As shown in Figure 4, we systematically include all possible dehydrogenation steps, where a hydrogen atom is removed from the adsorbate. We distinguish “normal” and deep dehydrogenations. As it takes two steps (two hydrogen atoms must be removed) to convert a single bond to a double bond, dehydrogenation reactions generally link a stable hydrocarbon and a monoradical (either as a reactant or as a product). Deep dehydrogenation reactions violate this rule, yielding multiple radicals. While they are generally unlikely on the reduced surface, the oxidized surface is so active that deep dehydrogenations readily proceed, occasionally surpassing normal dehydrogenation routes (for instance, CH_3_CH_2_ is preferentially deep-dehydrogenated to CH_3_CH instead of forming ethene). Intramolecular hydrogen migrations are omitted because their activation barriers exceed those of the dehydrogenation reactions.

**Figure 5:**
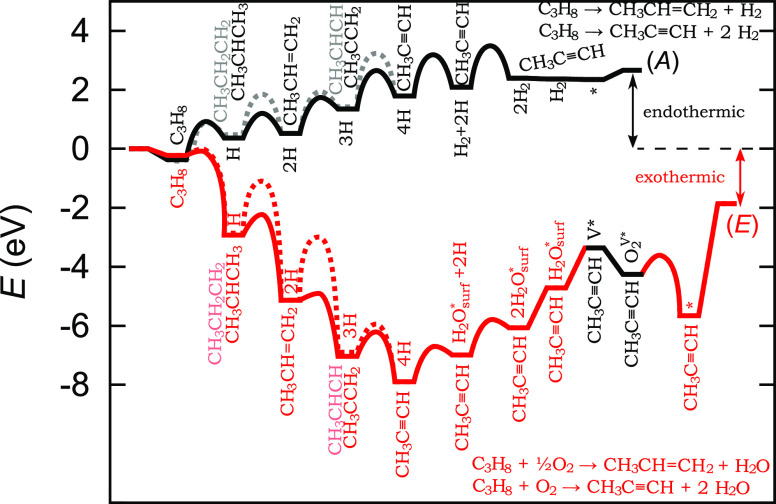
Potential energy surface for propane dehydrogenation over reduced (A) and oxidized (E) Cr_2_O_3_(0001). Deep dehydrogenations are not shown. Upon the removal of surface oxygen as H_2_O, the oxidized surface is reduced and reoxidized with O_2_ or N_2_O (latter not shown). Intermediates in light color and transition states connected with dashed lines are energetically disfavored.

Upon a weak physisorption of propane (0.36 eV on the reduced surface and 0.23 eV on the oxidized surface), the reaction proceeds rapidly on the oxidized surface. Due to very low barriers (*E*_A_ = 0.19 and 0.11 eV), both CH_3_CH_2_CH_2_ and CH_3_CHCH_3_ form. On the reduced surface, the barriers are much higher but nearly identical (*E*_A_ = 1.25 and 1.27 eV), meaning both intermediates also form, albeit much more slowly. However, on the reduced surface, CH_3_CHCH_3_ is more susceptible to further dehydrogenation (*E*_A_ = 0.84 eV), yielding propene. On the oxidized surface, CH_3_CHCH_3_ is dehydrogenated to propene (*E*_A_ = 0.69 eV), as expected, while CH_3_CH_2_CH_2_ undergoes deep dehydrogenation and yields CH_3_CH_2_CH instead (*E*_A_ = 0.55 eV). On the reduced surface, propene converts to propyne *via* CH_3_CCH_2_ (*E*_A_ = 1.22 and 1.31 eV). On the oxidized surface, CH_3_CH_2_CH is dehydrogenated to CH_3_CHCH (*E*_A_ = 0.21 eV) and propyne (*E*_A_ = 0.96 eV), while propene yields propyne *via* CH_3_CCH_2_ (*E*_A_ = 0.23 and 0.83 eV). Further dehydrogenation to CH_3_CC is possible on the reduced and especially oxidized surface (*E*_A_ = 0.92 and 0.95 eV, respectively), where it is exothermic. The potential energy surface of the reaction steps is depicted in Figure 5. In a nutshell, dehydrogenations are exothermic and kinetically more accessible on the oxidized surface and endothermic with higher barriers on the reduced surface.

Although C2 hydrocarbons enter the reaction as partially dehydrogenated species formed in various cracking reactions of the C3 intermediates, we model the entire pathway. On the reduced surface, ethene is formed in two steps (*E*_A_ = 1.42 and 1.42 eV), while on the oxidized surface CH_3_CH_2_ (*E*_A_ = 0.28 eV) preferentially dehydrogenates *via* CH_3_CH (*E*_A_ = 0.43 eV) and CH_2_CH (*E*_A_ = 0.13 eV) to ethyne directly (*E*_A_ = 0.13 eV). Consequently, very little ethene is formed. Methane is unlikely to dehydrogenate on the reduced surface, while the oxidized surface is conducive to full dehydrogenation to coke (C*), which has lower barriers and is exothermic. The ensuing C* cannot utilize adjacent surface oxygen atoms and desorb as CO or CO_2_ because of the strong endothermicity of such a reaction.

The increased reactivity of the oxidized surface is mirrored in much greater cracking activity as well. While on the reduced surface, most cracking reactions have high barriers (above 2.5 eV) and are very endothermic, on the oxidized surface, there are kinetically very accessible C–C cleavage reactions. For instance, CH_3_CH–C and CH_2_–C are exothermic and with barriers lower than 1.0 eV. C2 species fragment even more readily (CH_2_–C and C–C are especially prone to cleavage). On the reduced surface, fewer cracking reactions are accessible, although there are rare instances of reactions that occur on the reduced surface and *not* on the oxidized surface (CH_3_–CCH, CH_3_C–CH_3_, CH_3_–CCH_2_, and CH_3_–CH). As it will be shown later on, this greater activity of the oxidized surface manifests in both higher TOFs for the production of olefins and increased cracking, causing the formation of C2 and C1 products, and coking. The coke formed is usually burnt away with cycles of excess oxygen as CO_2_.

### Kinetic Modeling

#### Temperature Effect

Using no oxidant, the products of propane dehydrogenation are propene and hydrogen and the reaction is endothermic. When an oxidant is used, propene, CO_2_, side products (propyne, ethene, *etc.*), and H_2_O are produced in an exothermic reaction. On the reduced surface, the elementary step with the largest activation barrier is C_3_H_8_ → CH_3_CHCH_3_ + H (1.27 eV), while on the oxidized surface, that is, CH_3_CHCH_3_ → CH_3_CHCH_2_ + H (0.69 eV). However, on the oxidized surface, the formation of water (*E*_A_ = 1.19 eV) and its desorption (Δ*E* = + 1.36 eV) also play an important role.

**Figure 6:**
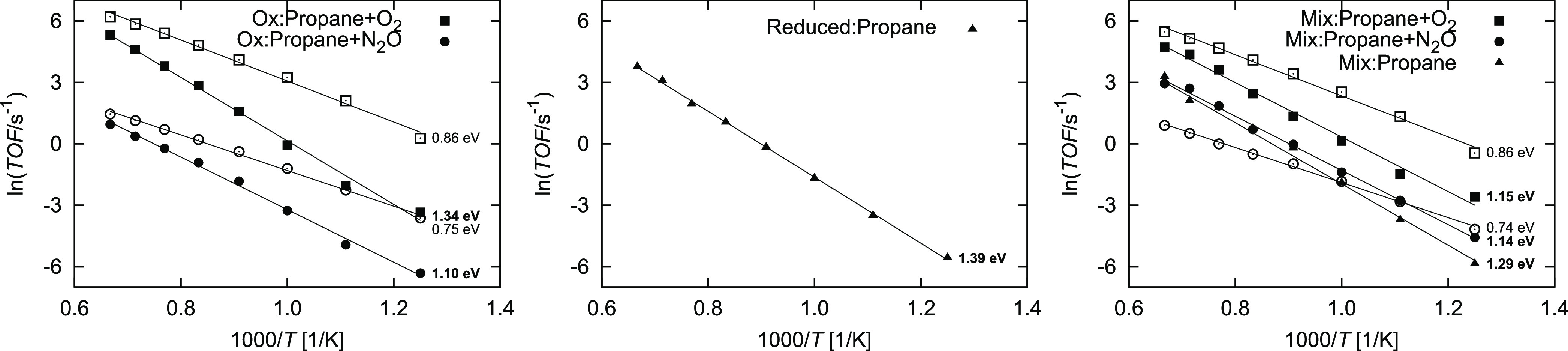
Arrhenius plots for the dehydrogenation of propane at *p*_C_3_H_8__ = 1.0 bar and *p*_oxidant_ = 1.0 bar on the oxidized (left), reduced (center), and mixed (right) surface. The mixed surface consists of 50% of each surface type and includes diffusion of the intermediates across the phase boundary. The symbol shape denotes the oxidant used: ■ O_2_, ● N_2_O, and ▲ none. Full symbols denote propene production, and empty symbols denote CO_2_ production. Inset energies correspond to the activation barriers.

The true apparent activation energies are shown in Figure 6. For the production of propene, this value is 1.34 eV with O_2_ and 1.10 eV with N_2_O. The larger value for O_2_ does not imply that the reaction proceeds slower, which is clearly shown in Figure 6. Instead, the larger value reflects a great temperature dependence, while the overall TOF is still larger. CO_2_ is produced with a higher TOF and lower temperature dependence (*E*_app_ = 0.86 and 0.75 eV with O_2_ and N_2_O, respectively). On the reduced surface (Figure 6), the apparent activation barrier is 1.39 eV and the effect of the oxidant is non-existent, which agrees with our previous work.^28^ We have also studied a mixed surface, consisting of an equal initial fraction of both types of active surface. As shown in Figure 6, the behavior of this surface is, as expected, between the both extrema. The apparent activation barrier for propene production is 1.15, 1.14, and 1.29 eV with O_2_, N_2_O, and no oxidant, respectively. When using O_2_, CO_2_ is still the main product, while with N_2_O, propene begins to predominate at lower temperatures.

A simple mathematical reasoning shows that the reactions with higher activation energies start to predominate at higher temperatures. However, it is known experimentally that at higher temperatures, hydrocarbons will convert to CO_2_ in oxidative conditions. This apparent inconsistency with our model is reconciled as follows. At higher temperatures, combustion proceeds homogeneously, while we are interested only in the performance of the catalyst, that is, surface reactions. Although the production of propene increases with temperature, this is in real life offset by homogeneous combustion. Second, real-life scenarios deal with significant catalyst deterioration at higher temperatures due to deactivation, coking, sintering, and so forth. While we include coking (*vide infra*), other transformations of the catalyst have not been included in the model because we are interested in the performance of the catalyst in its pristine reduced and oxidized forms and not in the description of the process *per se*.

#### Pressure Effect

**Figure 7:**
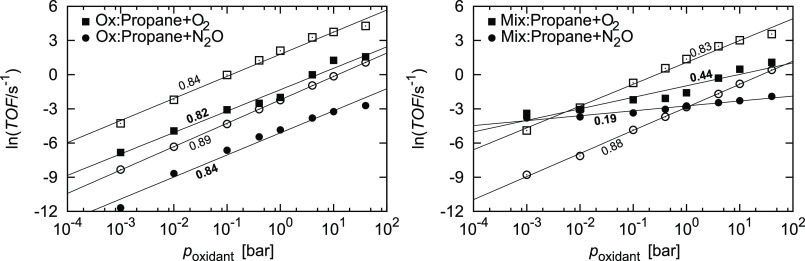
TOF for the dehydrogenation of propane at *p*_CH_3_CH_2_CH_3__ = 1.0 bar and *T* = 900 K and various oxidant types and pressures on the oxidized (left) and mixed (right) surface. The mixed surface consists of 50% of the oxidized and 50% of the reduced surfaces and includes diffusion of the intermediates across the phase boundary. The symbol shape denotes the oxidant used: ■ O_2_ and ● N_2_O. Full symbols denote propene production and empty symbols denote CO_2_ production. Inset values denote the slopes, which correspond to the reaction order with respect to the oxidant.

The effect of the oxidant used and its pressure on the reaction is shown in Figure 7. At 1 bar of propane and 900 K, the partial pressure of O_2_ and N_2_O was varied from 10^–3^ to 40 bar over the oxidized and reduced surfaces. On the former, increasing the partial pressure of the oxidant has little effect on the selectivity. The reaction order with respect to O_2_ and N_2_O is 0.82 and 0.84 for propene and 0.84 and 0.89 for CO_2_ production, respectively. On the mixed surface, the effect is different. Since CO_2_ production proceeds only on the oxidized surface, it is not surprising that the reaction order remains virtually unchanged. For the production of propene, the reaction order drops to 0.44 with respect to O_2_ and 0.19 with respect to N_2_O. In these more realistic conditions, the effect of oxidant pressure on the selectivity is predicted to have an important effect.

**Figure 8:**
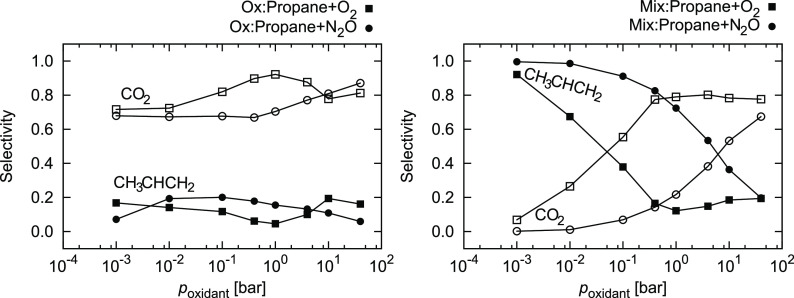
Selectivity toward CH_3_CHCH_2_ and CO_2_ in conditions as in Figure 7. Lines are guides for the eye.

This has clear implications for selectivity, which is shown in Figure 8. On the oxidized surface, the selectivity is poor, mostly below 20%, and does not change noticeably with oxidant pressure. On the mixed surface, the effect is pronounced. Whereas at lower oxidant pressures, the production of propene predominates, the selectivity precipitously drops at higher pressures. With oxygen, above 0.3 bar, selectivity toward CO_2_ plateaus at 80%. Using a softer oxidant yields consistently better selectivities, exceeding 90% below 0.1 bar N_2_O.

**Figure 9:**
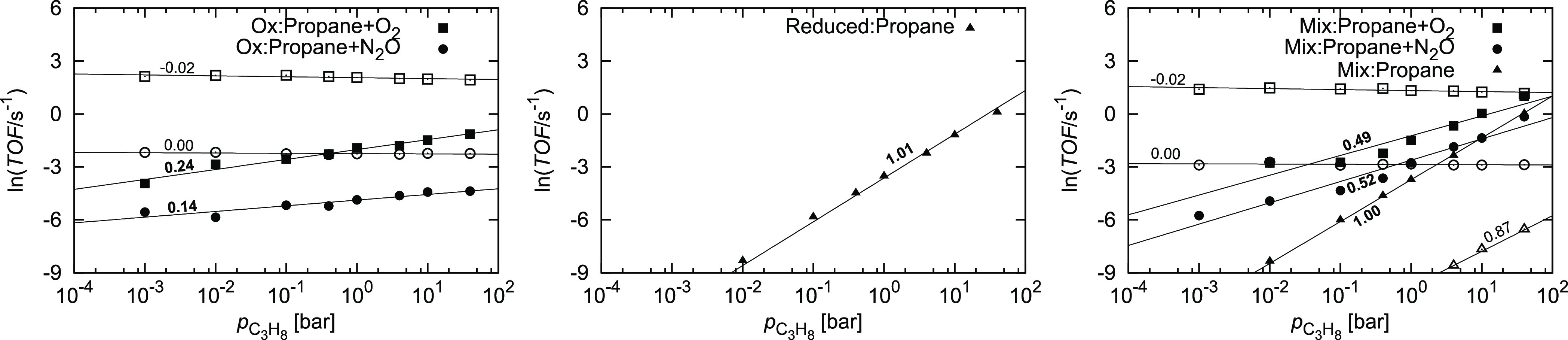
TOF for the dehydrogenation of propane at *p*_oxidant_ = 1.0 bar and *T* = 900 K and varying propane pressure on the oxidized (left), reduced (center), and mixed (right) surfaces. The mixed surface consists of 50% of oxidized and 50% of reduced surfaces and includes diffusion of the intermediates across the phase boundary. The symbol shape denotes the oxidant used: ■ O_2_, ● N_2_O, and ▲ none. Full symbols denote propene production and empty symbols denote CO_2_ production. Inset values denote the slopes, which correspond to the reaction order with respect to propane.

The influence of propane pressure was investigated at *p*_oxidant_ = 1.0 bar and *T* = 900 K and found to be converse (Figure 9). On the oxidized surface, the reaction order of propene production with respect to propane is 0.24 and 0.14 when using O_2_ and N_2_O, respectively, while the formation of CO_2_ shows a slightly negative reaction order with respect to propane. On the reduced surface, the reaction order with respect to propane is close to unity, consistent with our previous work.^28^ The behavior on the mixed surface falls between the two. For the production of CO_2_, the pressure of propane is irrelevant when using O_2_ or N_2_O as oxidants (without the oxidant, there is a positive trend with very low absolute TOF, which is attributed to the loss of surface oxygen atoms). For the production of propene, the reaction orders with respect to propane are 0.49, 0.52, and 1.00 with O_2_, N_2_O, and no oxidant, respectively. Higher propane pressures are thus expected to be advantageous for the production of propene.

**Figure 10:**
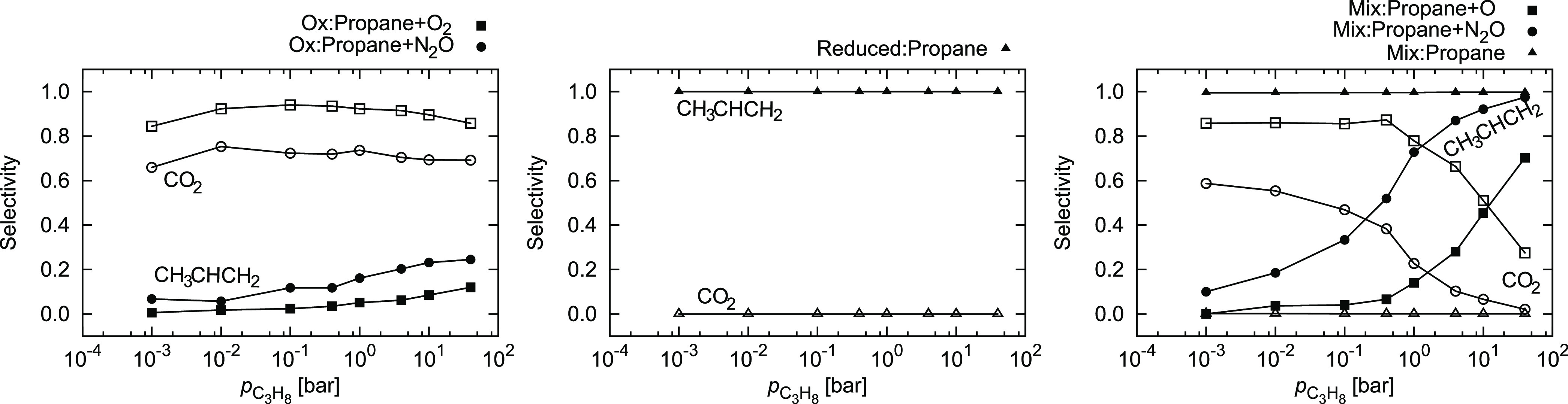
Selectivity toward CH_3_CHCH_2_ and CO_2_ in conditions as in Figure 9. Lines are guides for the eye.

In Figure 10, we show a stark difference between the performances of the three surfaces at different propane pressures. On the oxidized surface, the selectivity toward propene remains poor and only marginally improves even when the propane pressure shoots up. On the reduced surface, propane is almost exclusively produced. In the realistic conditions, which are modeled as a mixed catalyst, the selectivity increases with propane pressure from 10% below 0.001 bar to more than 90% above 10 bar. In all instances, N_2_O as a soft oxidant exhibits better selectivity, which is expected.

#### Surface Oxidation Effect

The analysis so far has focused on the effects of temperature and reactant pressure, which were tested on three arbitrary surface oxidation levels: fully oxidized, fully reduced, and mixed (50:50). While the oxidized surface exhibits high activity and low selectivity, the reduced surface has the exact opposite properties. Thus, a closer look into the optimum surface condition is warranted. We construct a series of mixed surfaces, as shown in Figure 1. The overall lattice size is 9 × 9 – there are three active sites per unit cell – where the bottom left corner (“island”) represents the oxidized portion of the surface. The size of the island was varied from 0 × 0 to 9 × 9 and kept fixed within the simulation run, probing different degrees of surface oxidation.

**Figure 11:**
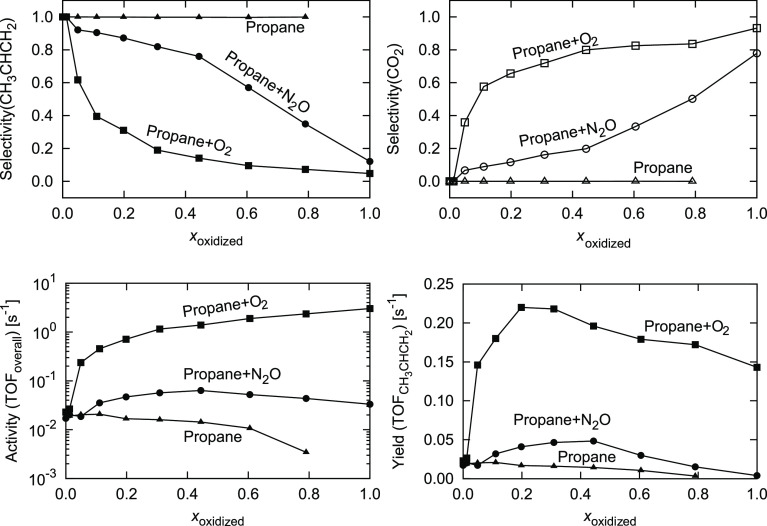
(Top) Selectivity toward propene (left) and CO_2_ (right), (bottom) catalyst activity (left) and propene yield (right). Propane dehydrogenation is performed at *p*_CH_3_CH_2_CH_3__ = 1.0 bar and *p*_oxidant_ = 1.0 bar at *T* = 900 K over surfaces with a varying fraction of oxidation. The symbol shape denotes the oxidant used: ■ O_2_, ● N_2_O, and ▲ none. Lines are guides for the eye.

In Figure 11, the performances of these surfaces at *p*_CH_3_CH_2_CH_3__ = 1.0 bar and *p*_oxidant_ = 1.0 bar at *T* = 900 K are shown. As expected, the selectivity toward propene drops as the degree of oxidation increases. The drop is more precipitous when using O_2_ as opposed to NO_2_. The selectivity toward CO_2_ displays a converse pattern because no other products are produced in any meaningful amounts; temperatures in excess of 1000 K were found to be required for the production of propyne and C_2_ products. However, the overall catalyst activity increases with the temperature. Thus, a maximum in propene yield was expected at some intermediate degree of oxidation. When using O_2_, the optimum degree of oxidation seems to be around 0.2, while with N_2_O the optimum lies around 0.5, but the effect is not as large.

**Figure 12:**
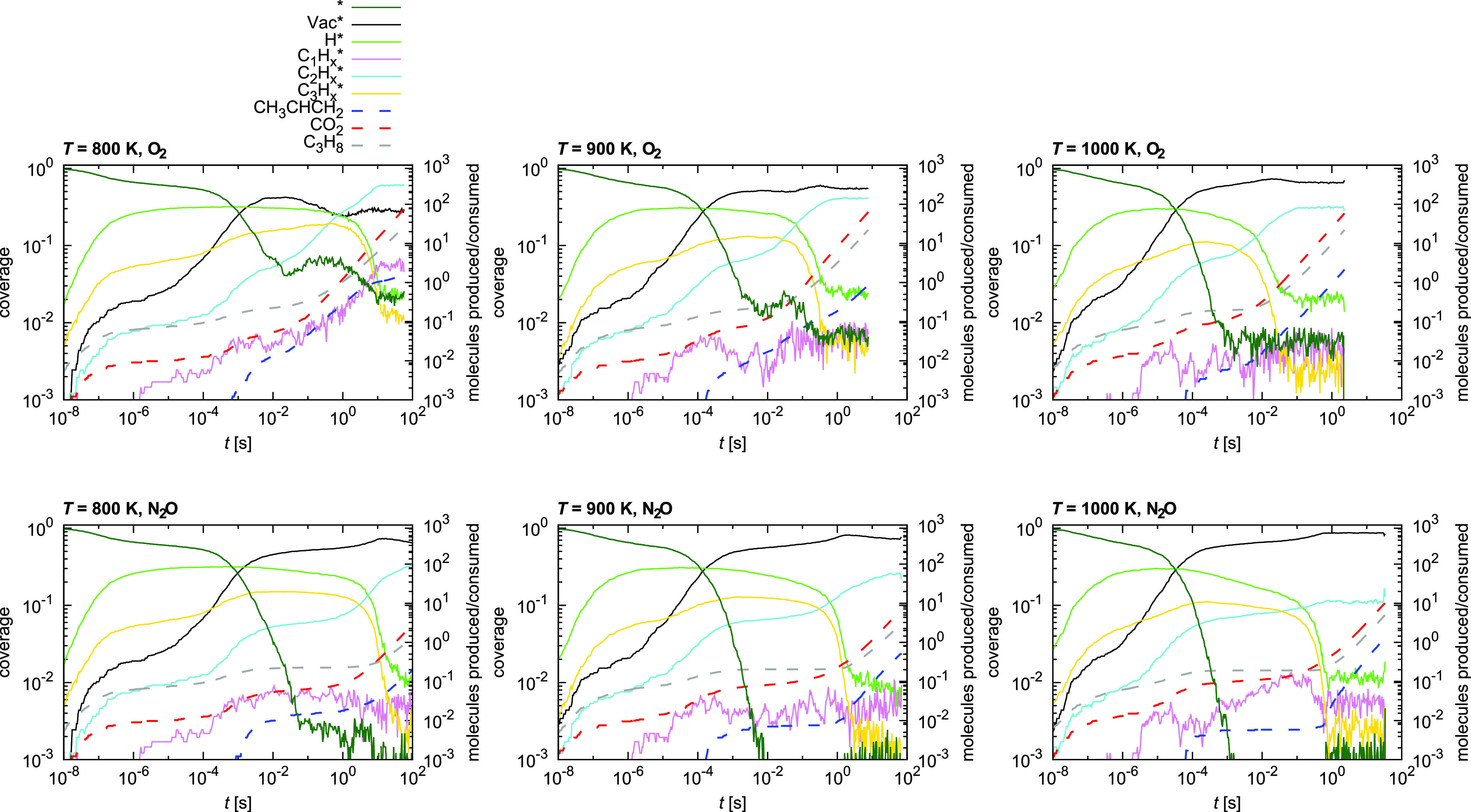
Coverage of the initially fully oxidized catalyst at *T* = 800 K (left), *T* = 900 K (center), and *T* = 1000 K (right), and *p*_CH_3_CH_2_CH_3__ = 1.0 bar and *p*_oxidant_ = 1.0 bar. The oxidant used is O_2_ (top) or N_2_O (bottom). Dashed lines represent the cumulative number of molecules produced (propene, CO_2_) or consumed (C_3_H_8_) *per active site* and use the right axis. The asterisk (*) represents empty oxidized sites and Vac* represents surface oxygen vacancies, that is, empty reduced sites.

For better insight into the catalyst performance, we study the catalyst surface, whose temporal evolution is shown in Figure 12. We plot the fraction of empty oxidized sites (*), surface oxygen vacancies (Vac*), adsorbed H*, and C_1_, C_2_, and C_3_ fragments at different temperatures. The rationale is as follows. The empty oxidized sites are readily available for the reaction. The surface oxygen vacancies actually correspond to the reduced catalyst surface, which is much less active (*vide supra*) and can be used as a surrogate for reversible catalyst inhibition. The adsorption of H* is transient and does not impede the reaction. Different carbon species, however, constitute irreversible deactivation of the catalyst. While C_3_ and most C_2_ species adsorb reversibly since they can undergo further dehydrogenation, the buildup of C_1_ is indicative of coking. The most abundant C_2_ species is CC*, which quickly plateaus and acts as an inhibitor. The C_1_ concentration steadily increases with time but can be burnt away with oxygen, regenerating the catalyst (in our model, we do not account for structural changes of the catalyst such treatment might cause). For comparison, the cumulative production of propene and CO_2_ and the consumption of propane is also shown on the secondary axes.

We observe a few clear trends. First, the fraction of empty oxidized sites decreases with temperature and is higher under O_2_ than under N_2_O, where it drops to zero. Conversely, the fraction of empty reduced sites increases and approaches unity at higher temperatures. In most instances, this is the predominant surface motif. The second most abundant species is initially H*, which predominates at low temperatures. The most common carbon species are C_2_^*^, followed by C_1_^*^, which is higher at lower temperatures. Once the reaction reaches a steady state, C_3_^*^ are negligible. These results explain the observed kinetic behavior of the catalyst under different conditions.

It is clear that under N_2_O, the surface is more reduced, which is beneficial for the selectivity toward propene. Moreover, we see that initially only CO_2_ is produced (red dashed line) and only when the catalyst is sufficiently reduced is propene also formed (blue dashed line). This transition to the steady state is also accompanied by the decrease of the surface concentration of H* and C_3_^*^.

#### Deactivation

Catalyst deactivation is a common problem with oxidation reactions. As coke forms on the catalyst surface, the activity drops. Deactivation is a complex topic, which a first-principles kinetic model cannot fully reproduce. We make no attempt at describing the structural deterioration of the surface, such as sintering, phase transitions, or decomposition. We define deactivation as the buildup of dead-end carbon species, which cannot further dehydrogenate even upon C–C cracking. These are: C*, CH*, CH_2_^*^, CH_3_^*^, CC*, CHC*, CH_2_C*, CH_3_C*, CH_3_CC*, CH_3_CHC*, and CH_3_CH_2_C*.

**Figure 13:**
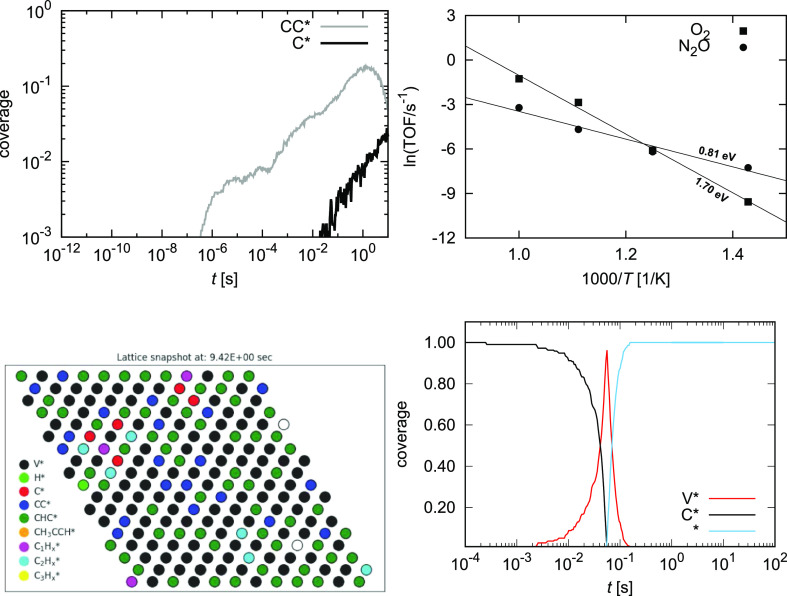
(Top left) Coverage of the catalyst with transient CC* and C* at *T* = 900 K and *p*_C3H8_ = *p*_O_2__ = 1 bar; (top right) the Arrhenius plot for the deactivation of the oxidized catalyst (measured as the buildup of CC* and C*) when using different oxidants (O_2_ and N_2_O); (bottom left) lattice snapshot after 9.42 s; (bottom right) regeneration of the coked catalyst with 1 bar O_2_ at 900 K when the C_3_H_8_ feed is cut off.

As shown in Figure 13, the deactivation of the catalyst occurs primarily by the accumulation of CC* and C* species. Other species (mostly CHC*) transiently form but are ultimately dehydrogenated. First, CC* is formed, which slowly transforms into C*. This might very well be an artifact of the model as both species describe the coked surface. Thus, we treat both species cumulatively in our analysis. We show that the deactivation of the catalyst can be described with the Arrhenius kinetics. The apparent activation barrier and pre-exponential factor for the deactivation are dependent on the oxidant used: 1.70 eV and *A* = 1.4 × 10^8^ s^–1^ for O_2_ and 0.81 eV *A* = 3.6 × 10^2^ s^–1^ for N_2_O. A much stronger temperature dependence when using a stronger oxidant is an expected result. We can compare these values with the previously calculated deactivation parameters on the reduced surface, which are *E*_A_ = 2.82 eV and *A* = 1.66 × 10^10^ s^–1^. This shows that the oxidized surface deactivates faster than the reduced surface when O_2_ is used as the oxidant. Soft oxidants, such as N_2_O, cause a much slower catalyst deactivation.

In Figure 13, we show the catalyst surface after 9.4 s. The catalyst surface is partially reduced (Vac*), while the oxidized sites are mostly coked (initially with CHC* and CC* and later with C*). When the catalyst is fully coked (Figure 13), it can be recovered by burning away the coke as CO_2_. Since C* is removed as CO_2_, the surface is briefly reduced and quickly reoxidizes. N_2_O and CO_2_ are too weak oxidants for surface recovery. In real operation, cycles of catalyst coking and regeneration repeat with an hourly cadence because the catalyst is not fully oxidized. As shown previously, the reduced surface gets coked much slower.^28^

### Experimental Relevance

It is difficult and often not very informative to try applying idealized models aimed at understanding the fundamentals in simplified conditions to real-life scenarios. Assumptions (pristine crystal lattice devoid of defects and phase boundaries, constant pressure and temperature, suppression of unwanted side reactions, reactor design, *etc*.) that make a model neat enough to discern trends can be next to impossible to ensure in experiments. However, some parallels can be drawn.

The experimentally measured adsorption energy of propane by Suzuki and Kaneko is 10 kcal mol^–1^ (0.43 eV), which is comparable with our values of 0.36 eV for the reduced surface and 0.23 eV for the oxidized surface.^19^ The apparent activation barrier for propane dehydrogenation in their study was found to be 33.9 kcal mol^–1^ (1.47 eV), which is comparable to values from our model (1.39 eV for the reduced surface and 1.34 eV for the oxidized surface).

Gascón *et al.* modeled the reaction of propane dehydrogenation over Cr_2_O_3_/Al_2_O_3_ with a lumped microkinetic model and found an activation barrier of (308 ± 14) kJ mol^–1^ (3.21 eV) for the reaction C_3_H_8_ → CH_4_ + C_2_H_4_.^24^ In our model, the decomposition of propane (C_3_H_8_ → CH_3_CH_2_ + CH_3_) has activation barriers of 3.23 and 3.02 eV on the reduced and oxidized surfaces, respectively. In the same model, the authors note that the “apparent reaction order of the coking process with respect to the propane concentration is very low”, which might be due to “the strong propene adsorption” and its coverage being close to unity. In our model, we also observe a weak dependence of the reaction rate on the propane concentration (see Figure 9) and very strong adsorption of propene on the oxidized surface (3.00 eV).

In their mathematical model of experimental data from the dehydrogenation of a propane-isobutane mixture in a fluidized bed reactor over Cr_2_O_3_/Al_2_O_3_, Vernikovskaya *et al.* obtained high selectivities toward isobutylene (89–94%) and propylene (81–100%).^63^ They decreased as the temperature increased, which is consistent with our model.

## Conclusions

We have investigated the mechanism and kinetics of propane dehydrogenation over oxidized and reduced α-Cr_2_O_3_(0001), which yields primarily propene and CO_2_. Other potential products, such as propyne and C2 and C1 hydrocarbons, could also form but only in minute amounts, especially when insufficient oxidant is present. We modeled the surface in the reduced and oxidized form. The reduced surface is Cr-terminated, while the oxidized surface has chromyl units exposed (−Cr=O). The kinetic parameters of all possible reaction steps were calculated using a first-principles DFT + *U* approach using the PW91 functional and D – *J* value of 5–1 eV. The mechanism was exhaustive, including all possible elementary steps for the dehydrogenation of propane to propene and propyne, cracking of hydrocarbons to C2 and C1 species and catalyst coking.

First, we show that the surfaces exhibit markedly different affinities toward the adsorbates. While saturated hydrocarbons and molecular hydrogen only physisorb on both surfaces, unsaturated intermediates bind much more strongly on the oxidized surface. On the oxidized surface, the reaction energy for the dissociative adsorption of hydrogen is −3.40 eV, while on the reduced surface, it is only −0.85 eV. Similarly, unsaturated C3 and C2 hydrocarbons have interaction strengths of 3–4 eV with the oxidized surface and 0.3–0.6 eV with the reduced surface. The difference stems from electronic effects, that is, surface basicity and nucleophilicity, as the oxidized surface exposes oxygen atoms with a Bader charge of −0.68*e*_0_, while the exposed Cr atoms on the reduced surface have a positive charge (+1.56*e*_0_).

As a consequence, the reaction mechanism is fundamentally different. This is to be expected from the stoichiometry alone as H_2_O is the co-product in the oxidative environment and H_2_ in the reducing environment. In the former case, dehydrogenation steps are exothermic with low activation barriers (generally below 1.0 eV), while the reduced surface exhibits higher barriers (typically 1–2 eV) and the steps are generally endothermic. Cracking reactions follow a similar trend, that is, being much more likely on the oxidized surface.

Dehydrogenation over the oxidized surface follows the Mars–van Krevelen mechanism. Hydrogen atoms that shed off the hydrocarbons are picked up by integral surface oxygen atoms, which desorb in the form of water. The ensuing surface oxygen vacancy corresponds to the reduced site. These are replenished with N_2_O, which heals one vacancy and yields N_2_, or O_2_, which donates two oxygen atoms. While both reactions are exothermic, N_2_O functions as a soft oxidant with a higher barrier. O_2_, however, first adsorbs without a barrier in a strongly exothermic step and then readily dissociates, making it a strong oxidant. The reaction with CO_2_ was calculated to be too endothermic on *this* surface and was not studied further.

Chang *et al.* have shown that the reduced surface with an additional oxygen vacancy is more active than the reduced surface.^20^ However, this structure was not predicted to be stable in their phase diagram by Wang *et al.*(50) Our calculations also showed that this further reducing of the reduced surface is energetically not favorable as the formation energy of such a vacancy is +4.07 eV (with respect to 1/2O_2_).

These data were cast into a KMC model, which also accounted for lateral interactions. It was shown that the reduced surface produces propene with high selectivity (>99%) but has a low activity. On the oxidized surface, the production of propene is approximately an order of magnitude faster. However, the production of CO_2_ is even faster, resulting in low selectivities. The reaction order with respect to propane is ≈1 on the reduced surface and 0.14–0.24 on the oxidized surface using O_2_ and N_2_O, respectively. On the oxidized surface, the reaction order is much more dependent on the oxidant pressure (≈0.8). The already low selectivity on the oxidized surface is further depressed if the oxidant pressure increases.

Realistic conditions during the reaction are between the two extrema (oxidized and reduced surface). First, the reaction was studied on the mixed surface, consisting of equal parts of both surface types. This surface exhibited considerably better performance than each individual surface. Additionally, the selectivity and activity were strongly dependent on the oxidant and propane pressure and oxidant type. Further investigation of surfaces with varying degrees of oxidation showed that there exists an optimum degree of surface oxidation, which is 0.2 when using O_2_ and 0.5 when using N_2_O.

We have shown that the catalyst also gets slowly deactivated due to the formation of carbon-heavy species. After the transient formation of CHC*, CH_2_^*^, CH*, CC*, and ultimately C* accumulate on the surface. The deactivation rate is strongly dependent on the temperature and the oxidant used. At higher temperatures with O_2_, the catalyst is very prone to coking, while with N_2_O at lower temperatures, it is less so. The formed coke can be removed by burning with O_2_, while N_2_O and CO_2_ are ineffective.

These results extend our knowledge of propane dehydrogenation on Cr_2_O_3_. While our previous work examined the reaction mechanism and kinetics on the reduced surface without co-feeding oxidants, which was shown to produce propene with high selectivity, here, we showed how the catalyst in question performs in more realistic conditions. The addition of the oxidant and consequent partial oxidation of the catalyst improves the activity and can be fine-tuned to avoid excessive CO_2_ production.
